# Surface modification of polyvinylidene fluoride (PVDF) membrane via radiation grafting: novel mechanisms underlying the interesting enhanced membrane performance

**DOI:** 10.1038/s41598-017-02605-3

**Published:** 2017-06-02

**Authors:** Liguo Shen, Shushu Feng, Jianxi Li, Jianrong Chen, Fengquan Li, Hongjun Lin, Genying Yu

**Affiliations:** 1 College of Geography and Environmental Sciences, Zhejiang Normal University, Jinhua, 321004 PR China; 2 Life Evaluation and Management Technology of Nonmetal Materials Lab, CGN National R and D Center, Suzhou, 215400 PR China

## Abstract

This study provided the first attempt of grafting hydrophobic polyvinylidene fluoride (PVDF) membrane with hydrophilic hydroxyethyl acrylate (HEA) monomer via a radiation grafting method. This grafted membrane showed an enhanced hydrophilicity (10° decrease of water contact angle), water content ratio, settling ability and wettability compared to the control membrane. Interestingly, filtration tests showed an improved dependence of water flux of the grafted membrane on the solution pH in the acidic stage. Atomic force microscopy (AFM) analysis provided *in-situ* evidence that the reduced surface pore size of the grafted membrane with the solution pH governed such a dependence. It was proposed that, the reduced surface pore size was caused by the swelling of the grafted chain matrix, with the pH increase due to the chemical potential change. It was found that the grafted membrane showed a lower relative flux decreasing rate than the control membrane. Moreover, flux of the bovine serum albumin (BSA) solution was noticeably larger than that of pure water for the grafted membrane. Higher BSA flux than water flux can be explained by the effects of electric double layer compression on the polymeric swelling. This study not only provided a pH-sensitive PVDF membrane potentially useful for various applications, but also proposed novel mechanisms underlying the enhanced performance of the grafted membrane.

## Introduction

While polyvinylidene fluoride (PVDF) is one of the most popular polymers used in the membrane industry and applications due to its distinct properties including high chemical resistance, good thermal stability, excellent processability and extraordinary mechanical properties^1–3^, the inherent hydrophobic characteristic of PVDF makes it susceptible to organic matter fouling^4–6^, which is always a precursor of bacterial infection, pore-blocking and foulant layer formation^7–9^.

In order to mitigate membrane fouling, numerous studies have been devoted to improve hydrophilicity of PVDF membranes by various physical and chemical methods^1, 5, 10, 11^. Many researchers have tried to simply blend polymeric membranes with the hydrophilic nanoparticles, such as nano metal oxides^12–15^, nano SiO_2_^16, 17^, carbon nanotubes^18, 19^ and graphene oxide (GO)^20, 21^. Although the operational conditions and cost of these methods are quite competitive, the drawbacks of aggregation and leaching of the nanoparticles largely limit the long-time antifouling performance of the organic/inorganic composite membranes. Compared with the physically blending method, the chemical grafting method can modify the membrane through chemical bonds, giving the membrane a long-time stable hydrophilic property^22–24^. Han *et al*.^25^ designed the PES composite membrane by the interfacial polymerization method and experimentally demonstrated that the sustainable antifouling performances were achieved even using real wastewater as the feeding. Tang *et al*.^6^ constructed the antifouling PVDF hollow fiber membrane via a zwitterionic graft copolymerization strategy and found that the irreversible fouling-induced resistance was prominently reduced from 53% to 15% due to the outstanding hydrophilicity of the prepared membrane. Through the grafting of certain functional groups, the contact angle of the membrane surface can be decreased to rather low values^26–29^. However, the involved complicated components in the grafting reaction, such as rigorous reaction conditions, initiating agents, catalysts and cleaning solvents, will undoubtedly increase the operational cost and may cause secondary pollution to the environment.

Fortunately, the high energy radiation grafting modification not only endows the membrane with a long-time stable performance, but also avoids over use of some expensive, poisonous chemicals and rigorous reaction conditions^30, 31^. Therefore, high energy radiation grafting modification has been widely conducted to obtain antifouling membranes in the literature. For example, Mok, *et al*.^32^ promoted the hydrophilicity of polyethersulfone (PES) hollow fiber ultrafiltration membranes by grafting polyethylene glycol (PEG) by γ-ray irradiation. The modified membrane exhibited a flux which is approximately 67% higher than the unmodified membrane during filtration of 50 ppm porcine albumin solution. Yang, *et al*.^33^ and our previous study^34^ respectively grafted N, N-dimethylacrylamide (DMAA) and N-vinyl pyrrolidone (NVP) onto PVDF powders by a γ ray pre-irradiation induced graft polymerization technique and then prepared filtration membranes via phase inversion method. The modified membrane performed enhanced hydrophilic properties which were evidenced by the reduced water contact angle. Besides, the degree of grafting can be well controlled by simply appropriately modifying irradiation absorbed doses. Moreover, another distinct advantage is that high energy radiation grafting modification can be applied easily in large-scale manufacturing. Hydroxyethyl acrylate (HEA) is an important functional monomer which gives hydroxyl functional groups to an acrylic polymer backbone. The hydroxyl groups act as crosslinking sites for hydrophilicity, improving resistance against corrosion and abrasion^11^. It has been widely used in the end applications including membrane modification^10, 35^. Although both PVDF and HEA are popular materials used for membrane technology, to our knowledge, neither study has explored grafting HEA onto PVDF membranes via radiation grafting methods. It is anticipated that such a combination may yield meritorious properties of the modified membrane. Moreover, the mechanisms underlying the performance of the modified membrane deserve intensive investigation.

In this study, Co^60^ was employed as the high-energy radiation source of γ ray to graft the hydrophilic HEA monomers onto the PVDF microfiltration membrane. The properties of the prepared PVDF-HEA membrane, especially the antifouling ability and the pH dependence of flux, were investigated through various designed experiments. The mechanisms underlying the properties of the grafted membrane were proposed. The findings obtained in this study offered deep insights into antifouling ability associated with grafting modifications.

## Materials and Methods

### Modification of membranes

**Figure 1:**
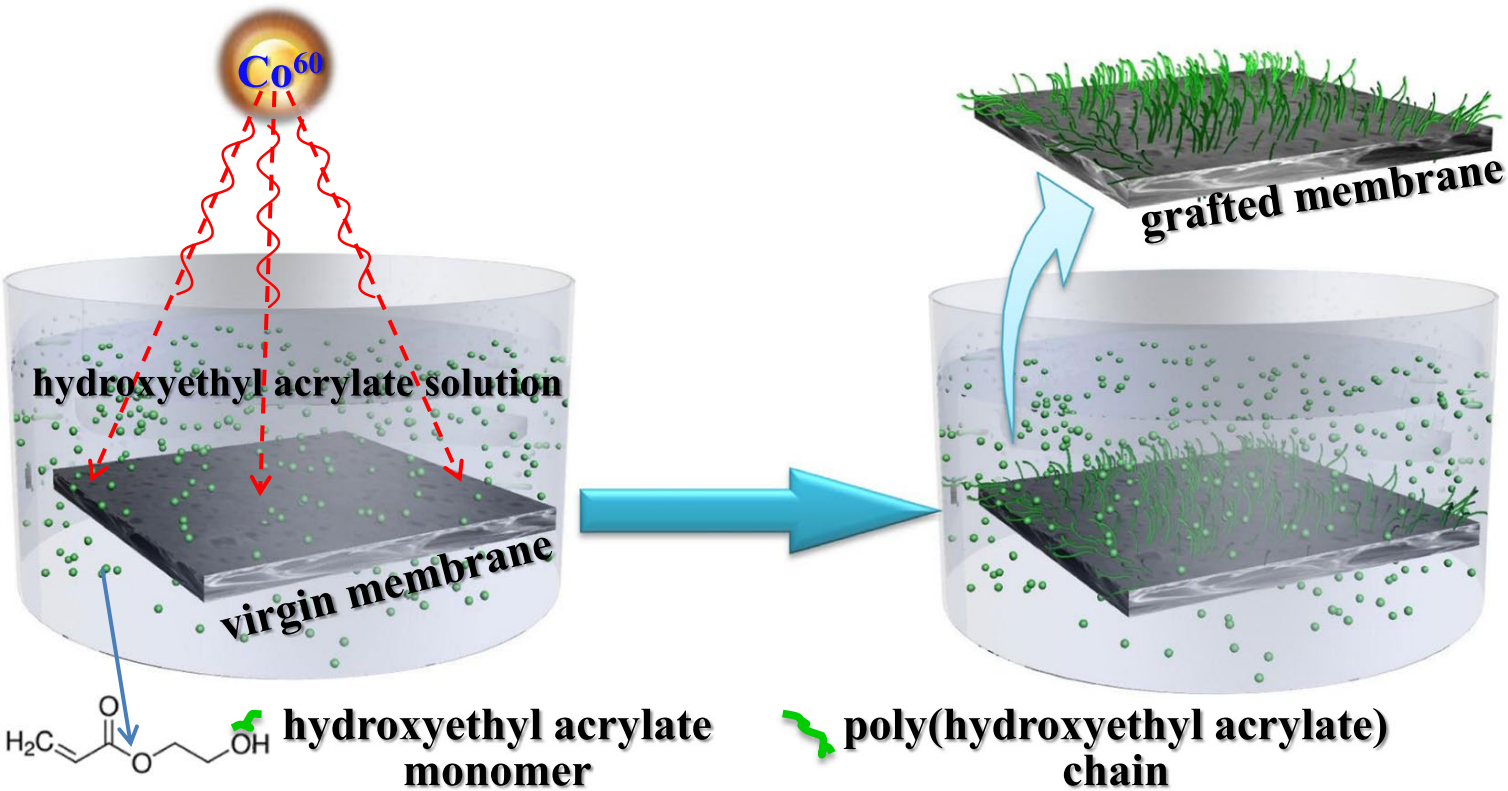
The schematic illustration of the grafting process.

The PVDF membrane (Pore size 0.1 μm, Jiangsu Dafu Co. Ltd.) was washed by pure water and then immersed into a glass tube which contains 5% wt of HEA (>99% wt, Sinopharm Chemical Reagent Co., Lt D, China) solution with 0.7% wt of ammonium ferrous sulfate (>99% wt, Sinopharm Chemical Reagent Co., Lt D, China) as the polymerization inhibitor. For grafting modification, studies have reported that the initiator concentration of 4–5% (w/v) was the optimum concentration to obtain better results^11, 36^. 5% HEA was adopted in this study for this reason. The glass tube was purged with nitrogen, and then irradiated with γ-rays from a Co^60^ source (Shanghai Institute of Applied Physics, Chinese Academy of Sciences, Shanghai, 201800, China) with a irradiation dose rate of 1.0 kGy/h at room temperature (25 ± 1 °C) for 20 hours. The grafting rate was calculated by the method reported in a previous study^37^. The grafted membrane was washed thoroughly and kept in pure water for testing. The schematic illustration of grafting process is shown in Fig. 1.

### Analytical methods

Fourier transform infrared (FTIR) spectroscopy using the attenuated total reflectance (ATR) mode was employed to study chemical alterations of the grafted membranes. The FTIR-ATR measurements were performed on a Perkin Elmer Spectrum 100 FTIR spectrometer with a resolution of 4 cm^−1^ and 32 scans per spectrum from 4000 to 400 cm^−1^.

Morphologies of membranes were measured by a scanning electron microscope (LEO1530vp SEM (Germany)). Samples were attached by carbon tape to the sample stage and sputtered with gold. The voltage was set at 25 kV and the current was set at 10 mA. The voltage of measurement was 20 kV.

A commercially available atomic force microscopy (AFM) instrument (Nanoscope 8, Bruker) equipped with a J scanner was used to observe the morphology of the membranes surface. AFM scanning was conducted in tapping mode with silicon nitride cantilevers (NP-S, Bruker). To observe the effects of different pH values on the structures of membrane surfaces, the samples were covered by a small Teflon container which was filled with solutions of different pH values. Then the small Teflon container was set up onto the objective table for testing.

The settling ability of the membranes was measured by a submerging experiment. In the operation, both the control and grafted membranes were cut into small pieces (approximately 0.5 cm × 2.0 cm) and then were transferred into the water surface. The water was sufficiently stirred for 10 min so that all the membrane pieces were randomly configured on the water surface. The submerging process was recorded at different time.

The contact angles of sludge and membrane samples were measured by a contact angle meter (Kino industry Co., Ltb, USA). Ultrapure water was chosen as the probe liquid in the experiments. A water drop (5 μL) was lowered onto the membrane’s surface from a needle tip. A magnified image of the droplet was recorded by a digital camera. Static contact angles were determined from these images with the calculation software. The contact-angle measurement was taken as the mean value of 5 different points on each membrane. For the contact angles of sludge foulants, the sludge is first filtrated on the membrane surface to form a cake layer and then kept in an oven for 24 hrs at 40 °C to remove water. In order to get a sludge plane, a glass plate was placed on it in order to flatten the sludge layer during the drying process.

Water content ratio measurements were conducted by a weight method which was similar with the previous method^14, 38^. All the samples were firstly dried at 50 °C for 24 h and then transferred into cups with pure water. After sitting for 12 h, the samples were taken out and dried for 24 h again at 60 °C. The membrane weights before (W_b_) and after (W_a_) drying were recorded for calculating the water content ratio as Eq. 1:

1 $${\rm{Water}}\,\,{\rm{content}}\,\,{\rm{ratio}}=\frac{{{\rm{W}}}_{{\rm{b}}}-{{\rm{W}}}_{{\rm{a}}}}{{{\rm{W}}}_{{\rm{a}}}}\times 100 \% $$

where, W_b_ and W_a_ are the membrane weights before and after drying, respectively.

### Filtration tests

**Figure 2:**
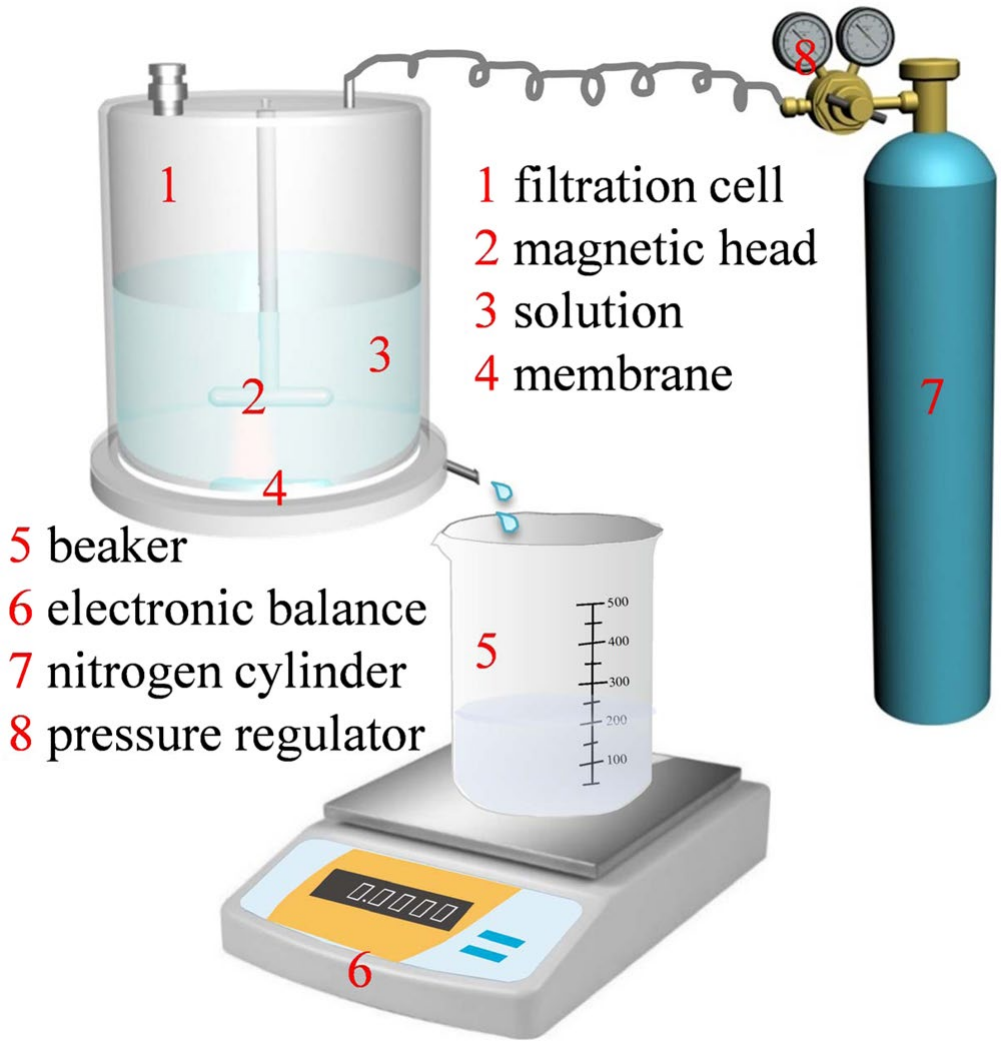
The schematic illustration of the set-up for filtration process.

The performance of the prepared membranes was analyzed through a previously adopted dead-end filtration system^39^. Figure 2 represents the scheme of the experimental filtration system. The valid membrane area in this system is 0.0012 m^2^, and the maximum volume of the filtration cell is 50 ml. The pressure of the measurement is 0.1 MPa supplied by a nitrogen cylinder. Bovine serum albumin (BSA) solution with 1.0 g/L concentration prepared by 0.1 M phosphate buffer solution with pH = 7.0 was used as the fouling probe solution. The antifouling property was measured by recording the flux decreasing and the flux decreasing rate during a continuous filtration process. The flux decreasing rate was determined using Eq. 2:

2 $${\rm{Flux}}\,{\rm{decreasing}}\,{\rm{rate}}=\frac{{{\rm{F}}}_{b}-{{\rm{F}}}_{e}}{[{\rm{Fi}}\,]\times {\rm{t}}}$$

where, F_b_ and F_e_ are the membrane fluxes at the beginning and end of the filtration process of BSA solution, respectively. [F_i_] is the value of the initial water fluxes for control and grafted membrane. Since the initial water fluxes of the control and grafted membrane are very different, the [F_i_] was introduced into the equation for a fair comparison. *t* is the time interval between beginning and end of the filtration process of BSA solution.

## Results and Discussion

### Surface characterization of the control and grafted membranes

#### FTIR analysis

**Figure 3:**
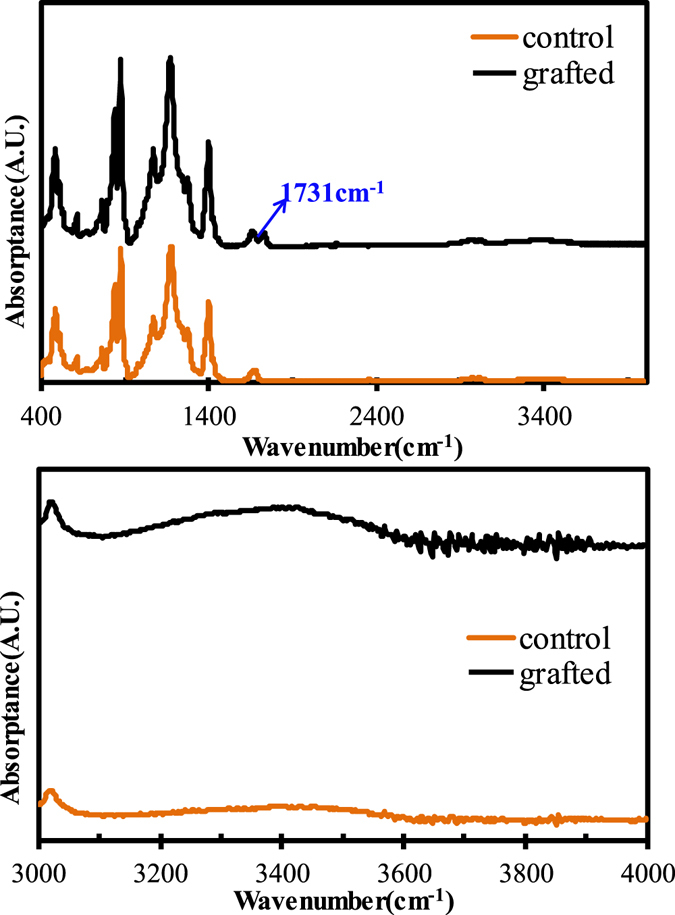
FTIR spectra profiles of the control and grafted membranes: (**a**) scan scope of 400 cm^−1^ −4000 cm^−1^; (**b**) the scan scope of 3000 cm^−1^ −4000 cm^−1^.

Figure 3 shows the FTIR spectra profiles of the control and grafted membranes. In Fig. 3(a), as compared with the spectra of the control (original) PVDF membrane, the grafted membrane spectra possess an obvious peak at the wavenumber of 1731 cm^−1^, which can be attributed to the characteristic peak of a C=O bond. It is not difficult to understand that the C=O functional groups are provided by the grafted HEA, indicating the success of grafting HEA on the membrane surface. According to the method reported in the literature^37^, the grafting rate was calculated to be 13.3%. In Fig. 3(b), the grafted PVDF membrane presents a higher IR responsive value of -OH functional group at the wide peak around 3300 cm^−1^ than that of the control PVDF membrane. A small wide peak of -OH functional groups in the FTIR spectra of the control PVDF membrane is presumably from the adsorption of a small amount of H_2_O molecules in air onto the membrane surface. While the extra responsive value of -OH functional groups at around 3300 cm^−1^ can be reasonably attributed to the contribution of the grafted HEA. It was reported that, the higher content of the hydrophilic -OH functional groups was able to efficiently predict the enhanced hydrophilicity and antifouling ability for the polymeric membranes^40^.

#### Morphology analysis

**Figure 4:**
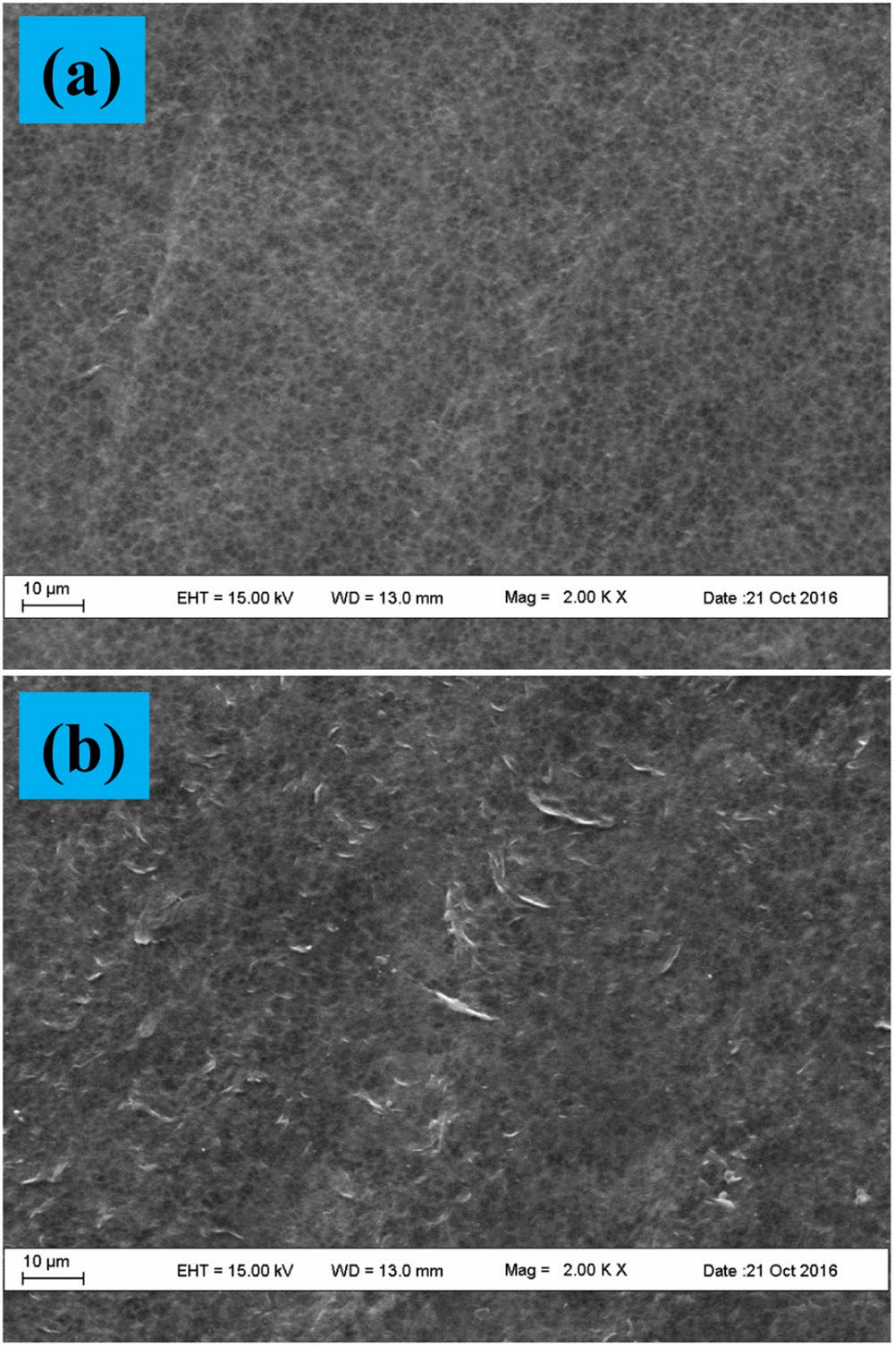
SEM images of the control and grafted membranes: (**a**) the control membrane; (**b**) the grafted membrane.

The surface morphologies of the control PVDF membrane and the grafted PVDF membrane were placed in Fig. 4. In Fig. 4, after the radiation grafting, the surface of PVDF membrane presented plenty of embossments (Fig. 4(b)) which indicated the polymerization of HA. It should be noted the absence of the original section membrane morphologies because the nonwoven supported PVDF membrane could not be quenched even in liquid nitrogen.

**Figure 5:**
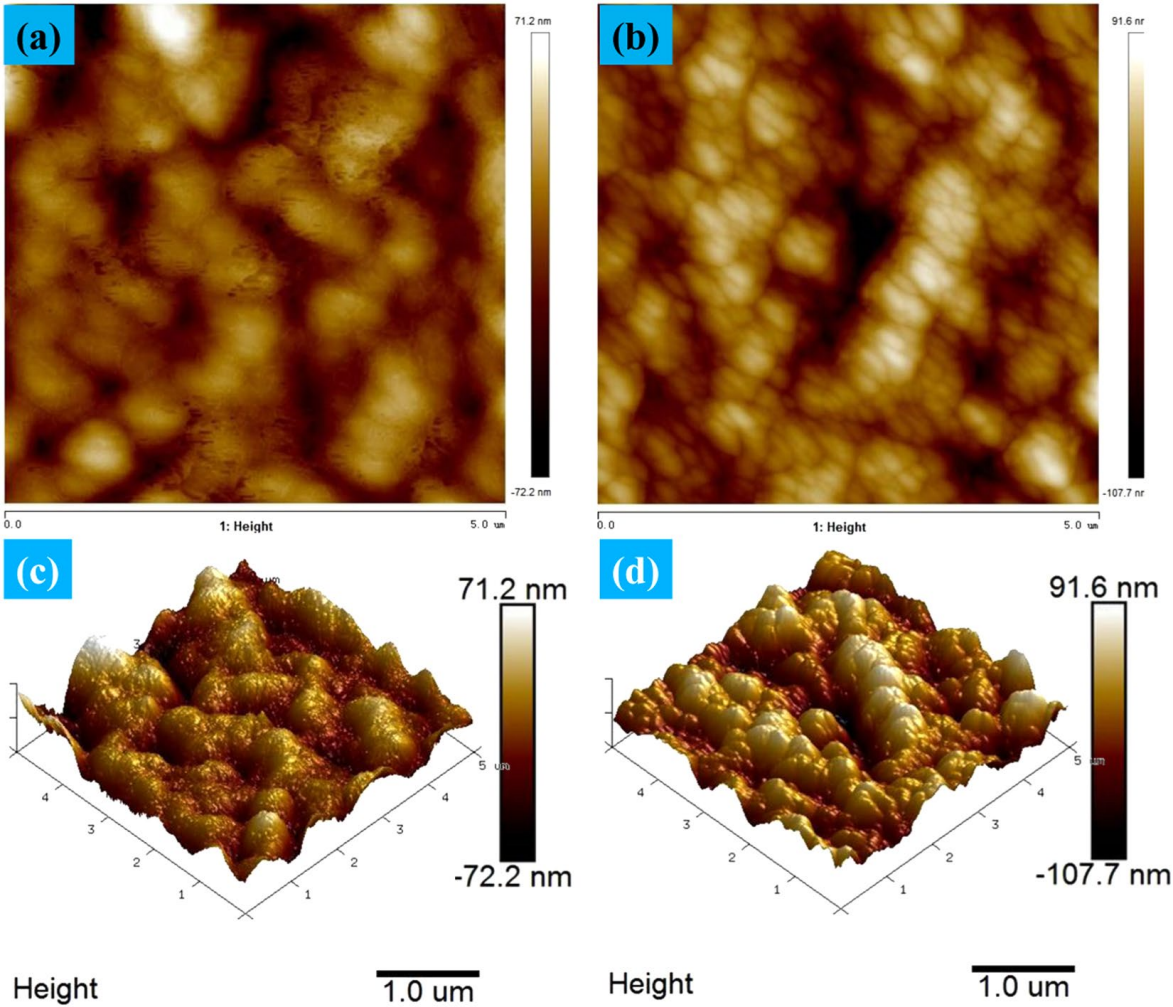
AFM images of the control and grafted membranes in top and perspective views: (**a**), (**c**) the control membrane; (**b**), (**d**) the grafted membrane.

More detailed information regarding membrane surface morphology can be represented by the AFM images in top and perspective views (Fig. 5). There exists obvious differences between the control and grafted membranes. Firstly, the grafted membrane surface demonstrates a subtler surface structure (Fig. 5(b) and (d)). Secondly, for the observed samples, the height fluctuation is ranged from −72.2 nm to 71.2 nm and from −107.7 nm to 91.6 nm for the control membrane and grafted membrane, respectively. In addition, the root mean square average roughness is 19.2 nm and 28.2 nm for the control membrane and grafted membrane, respectively. The AFM results show high consistency with the SEM observation (Fig. 4).

#### Settling ability, water contact angle and water content ratio

**Figure 6:**
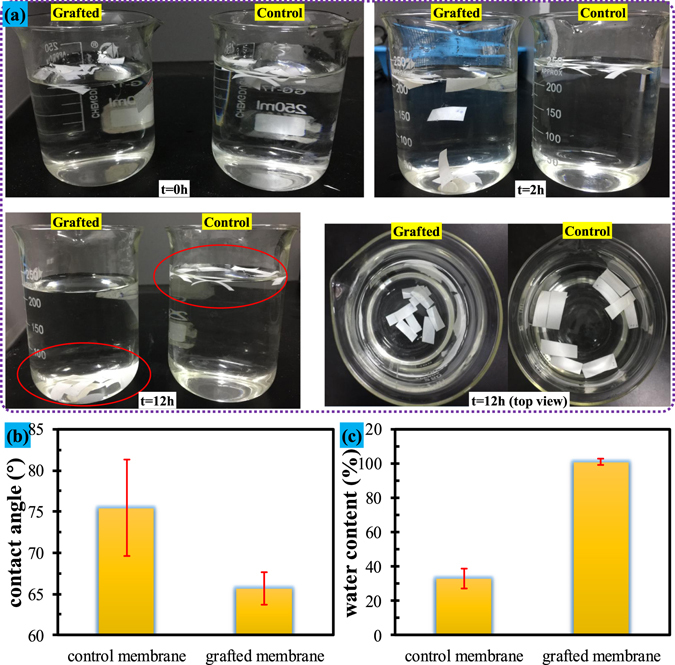
Settling ability, water contact angle and water content ratio of the control and grafted membranes: (**a**) settling ability; (**b**) water contact angle; (**c**) water content ratio.

Figure 6(a) shows the experimental procedure and results regarding settling ability of both control and grafted membranes. It can be seen that, at the beginning of the experiments, both the control and grafted membrane strips float on water. During the entire experimental duration, all the control membrane strips keep floating on water. In contrast, all the grafted membrane strips gradually settle down to the bottom of the water for up to 12 h. Figure 6(b) shows the water contact angle of both the control and grafted membrane. Comparing the counterpart of control membrane, the water contact angle of grafted membrane was reduced around 10 degrees, indicating an enhanced hydrophilicity of the grafted membrane. Meanwhile, water content ratio of the grafted membrane is 3 times higher than that of the control membrane (Fig. 6(c)), demonstrating a significantly improved wettability of the grafted membrane. The reason for the water residual for the control membrane was due to that the water was remained into the pore spaces. The reason for the water residual for the grafted membrane was because that the water was not only remained into the pore spaces but also held by the highly hydrophilic HEA. The enhanced hydrophilicity and wettability of the grafted membrane would well explain the improved settling ability as the grafted membrane is able to adsorb more water, and then increase its gravity. This characterization confirmed that grafting under conditions in this study can significantly improve hydrophilicity and wettability of the PVDF membrane.

#### Flux variation of control and grafted membranes under different pH values

**Figure 7:**
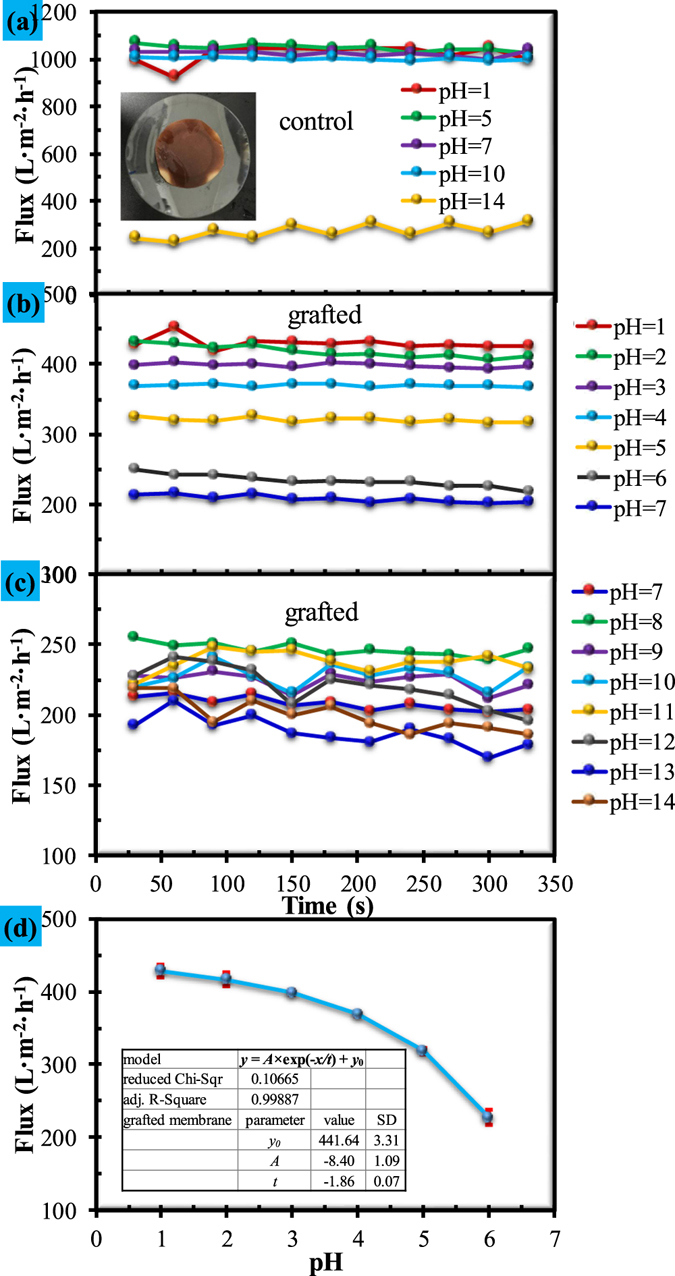
Flux variations of control and grafted membranes under different pH values: (**a**) control membrane flux at pH of 1, 5, 7, 10 and 14; (**b**) and (**c**) grafted membrane flux at pH of integer values in range of 1–14; (**d**) relationship between flux of the grafted membrane and pH values.

A series of solutions with pH of 1, 2, 3, 4, 5, 6, 7, 8, 9, 10, 11, 12, 13 and 14 were prepared according to pH adjusting strategy reported in a previous study^41^. Both the control and grafted membranes were subjected to filtration of this series of solutions, and the results are shown in Fig. 7. Figure 7(a) shows the flux variation with filtration time when filtered through a series of solutions with pH of 1, 5, 7, 10 and 14 for the control membranes. It can be seen that, there is no significant change in flux for all these solutions except for the solution with a pH of 14. The sudden decrease in flux for the solution with pH of 14 can be attributed to the severe corrosive effect of NaOH^42^, which is clearly illustrated by the inserted picture in Fig. 7(a). NaOH corrosion also influences the flux of the grafted membrane (Fig. 7(c)) where troublesome patters of flux in the base-stage can be observed. It is interestingly found that the flux of the grafted membrane demonstrates good dependence on the pH value in the acid-stage (Fig. 7(b)). In order to obtain a detailed relationship between membrane flux and pH value, the membrane flux in the acid-stage (pH 1 to 6) was plotted with the pH value, and the results are shown in Fig. 7(d). It can be seen that, the dependence of flux on the pH value for the grafted membrane can be described by an exponential equation with the *R* square of 0.99887 (inserted table in Fig. 7(d)).

**Figure 8:**
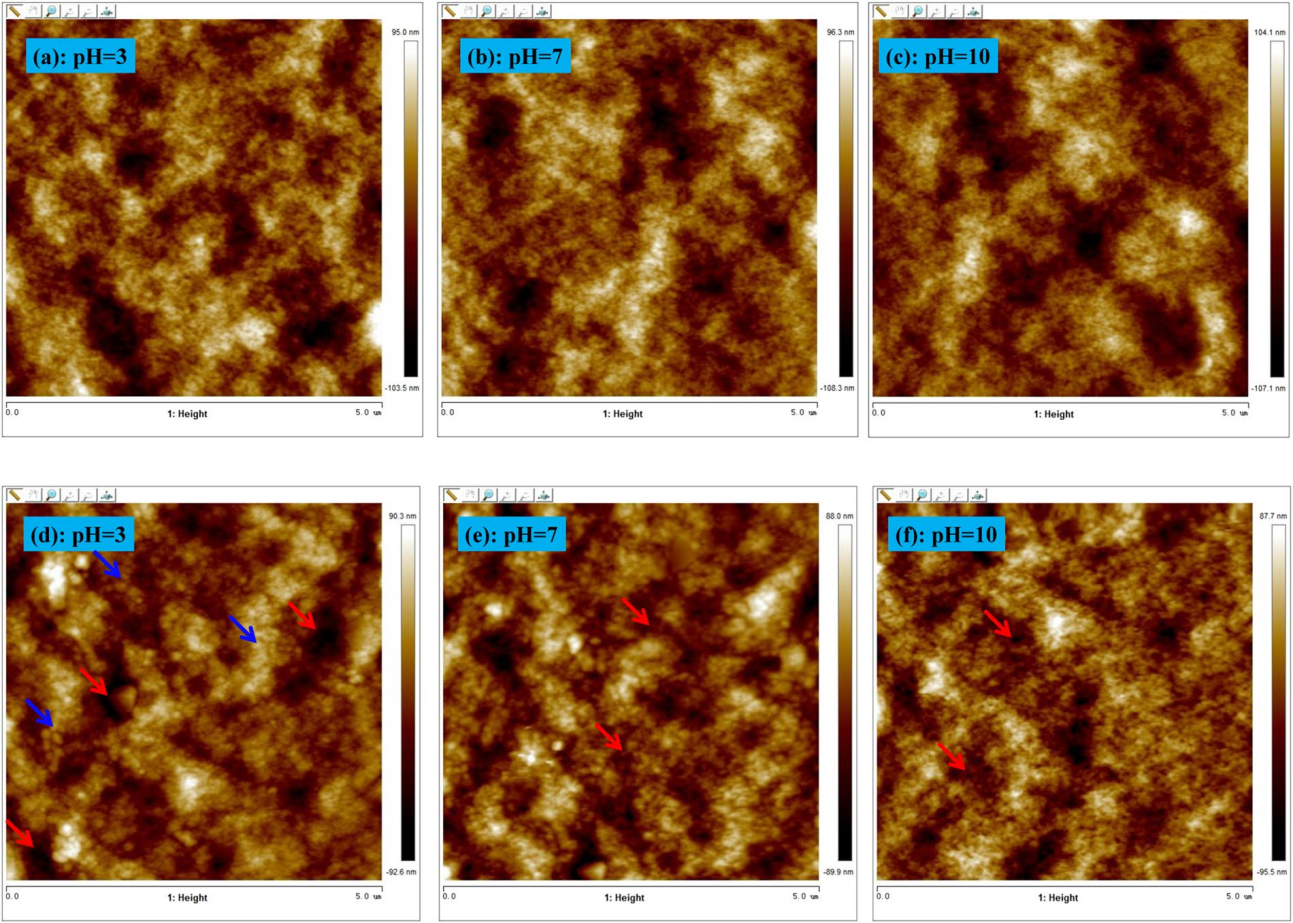
*In situ* surface topography variations of the control and grafted membranes under different pH values observed by AFM: (**a**–**c**) the control membrane under pH = 3, 7, 10, respectively; (**d**–**f**) the grafted membrane under pH = 3, 7, 10, respectively.

Experiments were conducted to explore the underlying cause of the interesting pH dependence property of the grafted membrane. Both the control and grafted membranes were set on the object stage of AFM and then measured under different solutions of pH values, and the AFM images are present in Fig. 8. As shown in Fig. 8(a–c), no obvious structural change happened on the control PVDF membrane when the pH increases from 3 to 7 and then, at 10. In contrast, for the grafted membrane, the surfaces show an embossment structure (blue arrows) in Fig. 8(d–f) as compared with Fig. 8(a–c). This is predictable because the grafting process will increase the roughness of the membrane surface. The increased surface roughness has been evident in Fig. 4 and Fig. 5. Moreover, Fig. 8(d–f) shows that the pore size in membrane surface decreases (red arrows) when solution pH increases from 3 to 7 and then, at 10. Therefore, the reduced flux of the grafted membrane with pH can be reasonably explained by the reduced pore size of the membrane surface. Figure 8 provides the *in-situ* experimental evidence of the mechanism underlying the pH dependence of flux of the grafted membrane.

**Figure 9:**
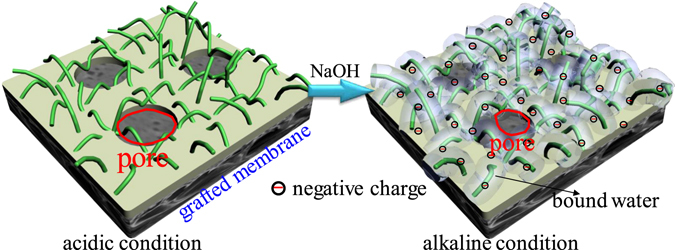
Schematic illustration of the configurational change of poly (HEA) chains grafted on membrane surface from acidic condition to alkaline condition.

The reason why solution pH can cause the reduced membrane surface pore size should reside in the surface chemical structure of the grafted membrane. Figure 9 shows the schematic illustration of configurational change of poly (HEA) chains grafted on membrane surface with solution pH. Hydrolysis of HEA would make available carboxyl and hydroxy groups contained in the grafted chains. Under strong acidic condition (low pH), carboxyl groups are protonated, causing a volume shrinkage of the chain matrix containing the carboxyl groups^43^. With the increase in pH, the grafted chain would be negatively charged due to the dissociation of carboxyl groups^44^. The experimental results from previous publications showed that the zeta potentials of polymers with carboxyl and hydroxy groups were highly dependent with the solution pH. For example, You *et al*.^45^ found that the zeta potential of poly(N,N-dimethylaminoethyl methacrylate (DMAEMA)/2-hydroxyethyl methacrylate (HEMA)) is slightly negative (−1.9 mV) at pH 6.8 and significantly decreased with the increase of solution pH. This was attributed to deprotonating of hydroxyl groups of HEMA. Moreover, Hu *et al*.^46^ reported that below pH 6, the zeta potential of the DMAEMA was positive and negative zeta potentials were obtained at pH above pH 6. Therefore, the present grafted membrane surface could be negatively charged when solution pH > 6, and the absolute value of zeta potential of the grafted membrane surface increased with the increase in pH range of 6–14. When the grafted membrane was subjected to filtration, there will be equivalent counter-ions present within the chain matrix for electro-neutrality of the whole system^47^. As a result, the chain matrix tends to swell (absorbing bound water), because in this way, the chemical potential of the whole matrix is lowest according to the physicochemical principles^48^. Figure 6 shows that the grafted membrane can absorb more water. Swelling of the chain matrix would reduce the pore size (Fig. 8). According to the physicochemical principles, the reduced membrane surface pore size with solution pH value is therefore plausibly explained.

This study actually provided a pH-sensitive PVDF membrane. Based on the configurational change of the grafted polymeric chains with solution pH, the surface pore size of membrane could be tunable, leading to regulation of the water flux. The pH-sensitive PVDF membrane can be potentially used for controlled drug release, control of water flux and pollutant rejection^43^. Considering the wide applications of pH-sensitive membranes, the distinct properties of PVDF materials, and the advantages of radiation grafting modification, it is argued that the findings obtained in this study are significant.

#### Antifouling performance of control and grafted membranes

**Figure 10:**
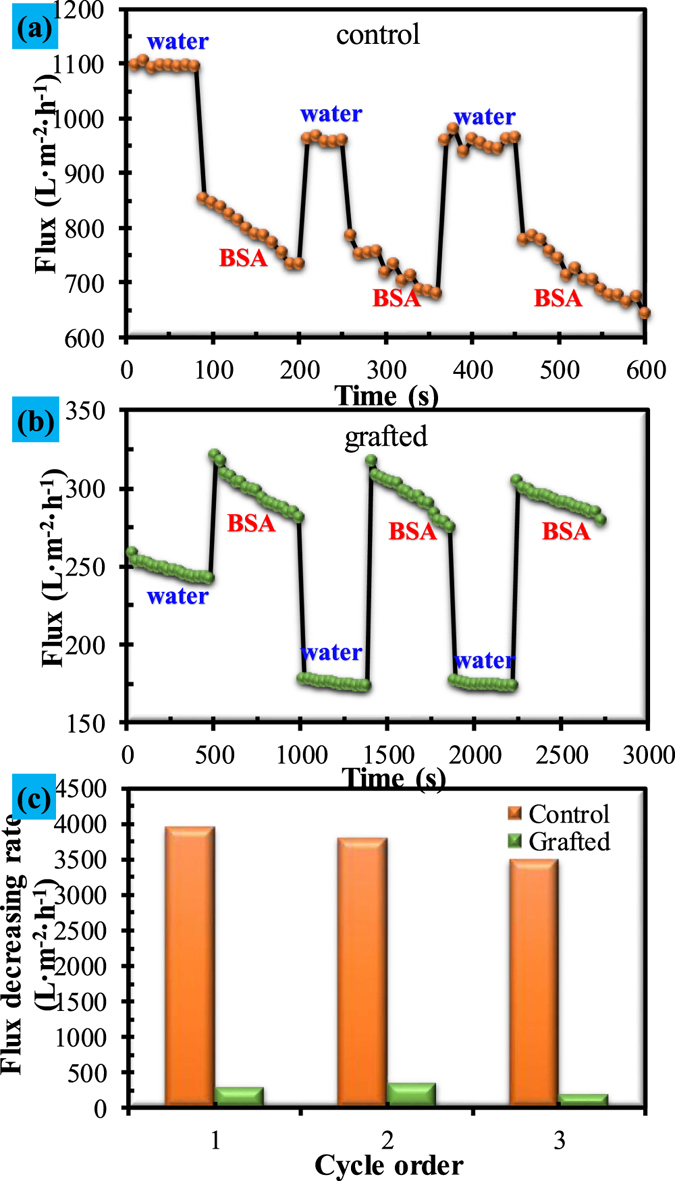
Antifouling performance of the control and grafted membranes: (**a**) flux of the control membrane by alternate filtration of pure water and BSA solution; (**b**) flux of the grafted membrane by alternate filtration of pure water and BSA solution; (**c**) flux decreasing rate of the control and grafted membranes.

In Fig. 10(a), the flux of the control membrane suffered a great loss (from 1094 L · m^2^ · h^−1^ to 678 L · m^2^ · h^−1^) when the water was replaced by BSA solution with the same pH value of 7.0. Thereafter, the flux loss augments continuously and reaches to around 60% after three cycles of filtration. The flux profile with filtration time indicates that the control membrane suffered severe membrane fouling within a short time of filtration. In contrast, the grafted membrane sensationally shows that the membrane flux of BSA solution was evidently larger than that of pure water in three whole cycles of filtration (Fig. 10(b)). The data indicating a decreasing flux rate in Fig. 10(c) clearly proved that the grafted membrane presented superior antifouling ability. It should be noted that, although the experimental result is sensational, it is reliable because the three cycles of filtration experiments conducted in triplicate showed the same situation.

Efforts have been made to explore the underlying cause of this phenomenon. In the literature, Kang, *et al*.^49^ grafted poly(2-hydroxyethyl methacrylate) (PHEMA) onto the porous surface of a polypropylene (PP) membrane, and found that the flux of the grafted membrane for the water and the BSA solutions decreased and increased with the increase in the grafting rate, respectively. It is expected that, if the grafting rate continuously increases, the BSA solution flux will exceed the water flux, providing a similar result to this study. Kang, *et al*.^49^ claimed that the increased flux of the grafted membranes for BSA solution was due to the reduced hydrophobic interaction between BSA molecules and the hydrophilic membrane surface. Such an explanation may be not clear enough to explain the interesting result in this study because hydrophobic interaction between water and the hydrophilic membrane should be extremely low.

**Figure 11:**
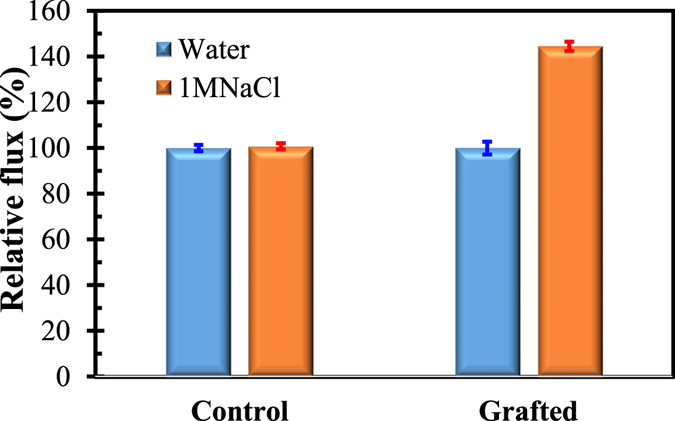
Effect of the ionic strength on the fluxes of the control and grafted membranes.

The exact mechanism underlying this phenomenon has yet to be explored. Figure 9 shows that the grafted membrane pore size is controlled by the swelling of the grafted chain matrix. Actually, according to physicochemical principles, swelling of the grafted chain matrix can be affected by either pH (Fig. 8) or ionic strength of solution. High ionic strength will lead to high electric double layer compression which will facilitate to resist swelling of the grafted chain matrix. Electric double layer compression can be expressed by the Debye length (λ), which is the thickness of the double layer that forms at the charged surface^50^. In polyelectrolyte solutions, Debye length can be considered as the distance within which the polyelectrolyte chain effectively carries the bound water^40^. This distance directly determines the extent of polyelectrolyte swelling. Debye length (λ) can be obtained by Eq. 3 ^51^.

3 $$\frac{1}{\lambda }=\sqrt{\frac{{e}^{2}\sum {n}_{i}{{z}_{i}}^{2}}{{\varepsilon }_{0}{\varepsilon }_{r}kT}}$$

where, *e* is the electron charge, $\sum {n}_{i}{{z}_{i}}^{2}$ is the ionic strength of solution, *n*_*i*_ is the number concentration of ion *i* in solution, *z*_*i*_ is valence of ion *i*, ${\varepsilon }_{r}{\varepsilon }_{0}$ is the permittivity of the solution, *k* is Boltzmann’s constant, and *T* is temperature. It can be seen that Debye length (λ) is inversely proportional to the square root of ionic strength of solution. When pure water is subjected to filtration, the grafted chain matrix will greatly swell due to the low ionic strength, which will significantly reduce the pore size. This mechanism can be verified by the low water flux of the grafted membrane (Fig. 10(b)) as compared with the high water flux of the control membrane (Fig. 10(a)). In this study, the BSA solution was prepared by dissolving BSA solid in 0.1 mol/L buffer solution. It was reported that Debye length would decrease from 9.64 nm to 0.964 nm when the ionic strength changes from 0.001 M to 0.1 M^40^. In Fig. 11, the flux of control membrane did not show obvious variation when the filtration liquid was replaced by1M NaCl, while the flux of grafted membrane was significantly affected by the 1 M NaCl solution which obviously promoted the flux (44.50%). Therefore, the relatively high ionic strength of the BSA solution will certainly resist swelling of the grafted chain matrix and enlarge surface pore size. As a result, BSA flux was observed to be higher than water flux of the grafted membrane (Fig. 10(b)). The proposed mechanism can reasonably explain the above interesting result for the grafted membrane.
